# Whole Genome Sequence Analysis of a Large Isoniazid-Resistant Tuberculosis Outbreak in London: A Retrospective Observational Study

**DOI:** 10.1371/journal.pmed.1002137

**Published:** 2016-10-04

**Authors:** Nicola Casali, Agnieszka Broda, Simon R. Harris, Julian Parkhill, Timothy Brown, Francis Drobniewski

**Affiliations:** 1 Department of Infectious Diseases and Immunity, Imperial College London, London, United Kingdom; 2 Centre for Immunology and Infectious Disease, Blizard Institute, Queen Mary University of London, London, United Kingdom; 3 Wellcome Trust Sanger Institute, Wellcome Trust Genome Campus, Hinxton, Cambridge, United Kingdom; 4 Public Health England National Mycobacterium Reference Laboratory, London, United Kingdom; 5 Departments of Microbiology and Respiratory Medicine, Barts Health NHS Trust, London, United Kingdom; University of California San Francisco, UNITED STATES

## Abstract

**Background:**

A large isoniazid-resistant tuberculosis outbreak centred on London, United Kingdom, has been ongoing since 1995. The aim of this study was to investigate the power and value of whole genome sequencing (WGS) to resolve the transmission network compared to current molecular strain typing approaches, including analysis of intra-host diversity within a specimen, across body sites, and over time, with identification of genetic factors underlying the epidemiological success of this cluster.

**Methods and Findings:**

We sequenced 344 outbreak isolates from individual patients collected over 14 y (2 February 1998–22 June 2012). This demonstrated that 96 (27.9%) were indistinguishable, and only one differed from this major clone by more than five single nucleotide polymorphisms (SNPs). The maximum number of SNPs between any pair of isolates was nine SNPs, and the modal distance between isolates was two SNPs. WGS was able to reveal the direction of transmission of tuberculosis in 16 cases within the outbreak (4.7%), including within a multidrug-resistant cluster that carried a rare *rpoB* mutation associated with rifampicin resistance. Eleven longitudinal pairs of patient pulmonary isolates collected up to 48 mo apart differed from each other by between zero and four SNPs. Extrapulmonary dissemination resulted in acquisition of a SNP in two of five cases. WGS analysis of 27 individual colonies cultured from a single patient specimen revealed ten loci differed amongst them, with a maximum distance between any pair of six SNPs. A limitation of this study, as in previous studies, is that indels and SNPs in repetitive regions were not assessed due to the difficulty in reliably determining this variation.

**Conclusions:**

Our study suggests that (1) certain paradigms need to be revised, such as the 12 SNP distance as the gold standard upper threshold to identify plausible transmissions; (2) WGS technology is helpful to rule out the possibility of direct transmission when isolates are separated by a substantial number of SNPs; (3) the concept of a transmission chain or network may not be useful in institutional or household settings; (4) the practice of isolating single colonies prior to sequencing is likely to lead to an overestimation of the number of SNPs between cases resulting from direct transmission; and (5) despite appreciable genomic diversity within a host, transmission of tuberculosis rarely results in minority variants becoming dominant. Thus, whilst WGS provided some increased resolution over variable number tandem repeat (VNTR)-based clustering, it was insufficient for inferring transmission in the majority of cases.

## Abstract 2

In this retrospective observational study, Francis Drobniewski and colleagues assess the power and value of whole genome sequencing to resolve transmission network of a large tuberculosis outbreak.

## Author Summary

**Why Was the Study Done?:**

- Whole genome sequencing (WGS) of pathogenic organisms, such as the bacterium that causes tuberculosis, is increasingly used to better understand bacterial diversity, evolution, and drug resistance, and transmission to humans.
- WGS is believed to be superior in defining the extent and direction of tuberculosis transmission compared to the main existing standard method (variable number tandem repeat [VNTR] analysis).
- Interpretation rules have been proposed using a 12 single nucleotide polymorphisms (SNP, or single point changes in DNA sequence) distance as the upper threshold to identify plausible transmissions and argue that isolates separated by five or fewer SNPs are likely to have resulted from recent transmission; but the extent of variation amongst different bacteria in the same patient (intra-host variation) and the real value of WGS in analysing larger VNTR-defined outbreaks has received little attention.

**What Did We Do and Find?:**

- The study focused on understanding the transmission of tuberculosis strains resistant to one of the main therapeutic drugs (isoniazid) in an outbreak centred on London, England, from 1998 to 2012.
- WGS was performed on 344 strains, with 96 shown to be indistinguishable, 105 differing by a single SNP, and only one isolate differing from the major clone by more than five SNPs. Overall, only 270 SNPs discriminated isolates within the cluster.
- WGS was used to investigate intra-host strain variation, demonstrating between zero and three SNPs difference in six pairs of patient isolates obtained from pulmonary sites 9 to 35 mo apart, with up to a seven SNP variation in multiple single bacterial colonies isolated from the same patient.

**What Do the Findings Mean?:**

- WGS provides increased resolution over VNTR-based clustering, but it is insufficient to fully resolve chains of transmission, as multiple transmission events can occur with no detectable SNP acquisition, and even identical isolate pairs cannot be proven to have resulted from a recent transmission event without supporting epidemiological evidence.
- Centralized routine WGS of TB strains for routine surveillance/outbreak investigation may be misleading and misdirect public health action without additional epidemiological evidence.

## Introduction

A cluster of isoniazid-monoresistant tuberculosis cases centred on North London was initially detected in 2000 [1]. Molecular typing based on IS*6110* restriction fragment length polymorphism (RFLP) analysis confirmed the isolates comprised a putative outbreak, and retrospective investigation revealed that the first cluster case probably occurred in 1995. RFLP-based typing of isoniazid-monoresistant isolates and, later, routine 24-loci mycobacterial interspersed repetitive units-variable number tandem repeats (MIRU-VNTR) typing showed that by 2008, there were 343 culture-proven cases, 299 (87%) of which were diagnosed in London [2]. By the end of 2013, the cluster comprised 501 individuals nationally [3]. Compared to other tuberculosis cases in London, outbreak cases were more likely to be United Kingdom born (53%) and of white or black Caribbean ethnicity. Patients were from a wide social background with foci in high risk groups, including the homeless, injection-drug users, and prisoners [1,4,5].

Several lines of evidence suggested that the outbreak strain was highly transmissible. Epidemiological investigations revealed that some of the individuals became infected after relatively brief periods of patient contact [1], and there were more than twice as many smear-positive patients amongst outbreak cases compared to other London cases (52% versus 18%) [4]. Screening of social and household contacts of the first 100 cases identified found relatively high overall rates of transmission, amounting to 11.3% of contacts (compared to an average of 1% for other documented outbreaks), whilst transmission to close contacts of smear-positive cases reached 21.5% [6].

The aim of this study was to investigate the power of whole genome sequencing (WGS) to resolve the transmission network within the London isoniazid-resistant tuberculosis outbreak and to determine the value of WGS compared to 24-loci MIRU-VNTR–based clustering and traditional epidemiological investigations based on contact tracing. Interpretation of the phylogenetic relationships between patient isolates depends on understanding the extent of diversity in the background population, the diversity within an infected host, and the population bottleneck associated with transmission events [7]. Thus, we sought to provide context for the outbreak analysis by investigating intra-host diversity within a specimen, across body sites, and over time. Additionally, clustered isolates were compared to closely related non-outbreak isolates. Finally, we sought to identify genetic factors leading to the epidemiological success of this cluster.

## Methods

### Case Identification and Isolate Selection

Since 2000, the Health Protection Agency (now Public Health England) National Mycobacterium Reference Laboratory has identified *Mycobacterium tuberculosis* isolates belonging to the outbreak through molecular fingerprinting as part of its regional and national public health function. Clustering was based initially on IS6110 RFLP analysis, later by a PCR-based screening method (“rapid epidemiological typing,” [RAPET]), and, since 2010, by routine 24-loci MIRU-VNTR typing. The outbreak profile corresponds to Public Health England (PHE) cluster E1244 (424332431515321236423–52; including an untypeable 3690 locus).

In order to compare between-host to within-host diversity, we selected pairs of isolates from patients that were (1) isolated from pulmonary sites more than 6 mo apart, (2) isolated from a pulmonary and extra-pulmonary site, or (3) acquired an additional drug-resistance phenotype. In addition, we isolated 27 individual colonies from a single sputum specimen and sequenced each.

A further 12 related isolates that differed from the E1244 profile at one or more loci were selected for sequencing to compare WGS-based clustering with MIRU-VNTR–based clustering.

The prospective analysis plan is provided in S1 Text. As this study assessed utilised routine surveillance, diagnostic, and molecular epidemiological data available to the National Mycobacterial Reference Laboratory within the public health service, and as no sequencing results were utilised for clinical management, research ethics committee approval was not required in the UK. Therefore, patient consent was not required or obtained.

### Molecular Fingerprinting and Microbiological Methods

RFLP, based on the insertion sequence IS*6110*, was performed as previously described [8]. MIRU-VNTR typing was performed with a panel of 24 loci using a high-throughput method based on multiplex PCR with fluorescent-labelled primers followed by fragment separation on a CEQ8000 Genome Instrument (Beckman Coulter) [9]. Spoligotyping was conducted according to standard methods [10].

Phenotypic drug susceptibility testing against isoniazid, rifampicin, ethambutol, and streptomycin was performed using the resistance ratio method on Löwenstein–Jensen medium [11]. Pyrazinamide susceptibility was determined in biphasic media [11].

### Whole Genome Sequencing

Aliquots of archived *M*. *tuberculosis* cultures were inoculated onto Middlebrook 7H11 agar supplemented with 10% OADC and grown to near confluence. Cultures were harvested, resuspended in Tris-EDTA, and heat-killed. Bacilli were lysed by vortexing with 0.1 mm glass beads. Genomic DNA was purified from the cleared lysate using a DNeasy Blood and Tissue kit (Qiagen). Multiplexed libraries with a 200-bp insert size were prepared and subjected to paired-end sequencing with a readlength of 100 bp on an Illumina HiSeq at the Wellcome Trust Sanger Institute. Raw data were deposited in the European Nucleotide Archive (www.ebi.ac.uk/ena) with accession number ERP003508.

### Data Analysis

Sequencing reads were mapped to a corrected version of the *M*. *tuberculosis* H37Rv reference sequence [12] using SMALT [13]. Mean mapping depth was 84-fold (37- to 126-fold). Candidate SNPs were identified using SAMtools [14]. At each mapped position, alleles were considered valid if supported by at least two and greater than 70% of mapped reads on each strand, with a minimum mapping quality of 45. SNPs were excluded from further analysis if they were located within repetitive regions, including the PE-PPE genes, or if they did not discriminate within the cluster. Genome positions with missing genotypes in more than half of all isolates were also excluded.

For creating minimum spanning trees (MSTs), remaining ambiguous basecalls (379/153874; 0.04%) were assigned to wild type. MSTs were calculated by BioNumerics v7.5 (Applied Maths), allowing creation of hypothetical nodes. Maximum-likelihood phylogenies were reconstructed with RAxML using a general time-reversible model with gamma correction for among-site rate variation [15] and visualised with FigTree [16].

The impact of including variation within the PE-PPE gene family (168 genes covering 6.4% of the genome) was assessed. SNPs at 49 genomic loci passed quality and exclusion filters. The proportion of ambiguous basecalls at these loci was 3.9% (780/19894 bases), which was 100 times higher than the non-repetitive region of the genome included in this analysis (0.04%, 379/153874), supporting the decision to exclude these genes.

Pindel [17] was used to detect indels and structural variants, which were verified by viewing mapping files in Artemis [18]. SpolPred [19] was used to predict spoligotypes from sequencing reads.

## Results

### Study Population

**Fig 1:**
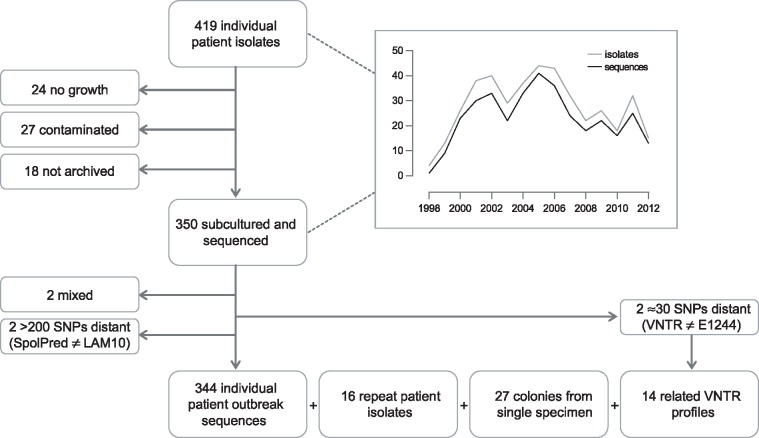
Flow diagram of sample selection. Graph depicts number of isolates/sequences included by year, 1998–2012.

Between 2 February 1998 and 22 June 2012, the UK National Mycobacterium Reference Laboratory received isolates from 419 individual patients that were associated with the outbreak due to sharing a molecular type. Isolates from 350 patients were successfully subcultured and sequenced (Fig 1). Although not all outbreak strains were successfully re-cultured, there did not appear to be any bias between the group of strains that were cultured and sequenced and those that were not cultivable. There was no bias in when the isolates were collected (Fig 1), and there was no statistical difference between the two groups in regard to those patients who were children or adults (Fisher exact test, *p* = 0.62), those with pulmonary compared to extrapulmonary disease (chi-square test, *p* = 0.77), and whether there was any additional resistance or just isoniazid resistance (Fisher exact test, *p* = 0.70).

Preliminary sequence analysis resulted in the exclusion of two isolates that were mixed. In silico spoligotype prediction indicated that two isolates, separated by more than 200 SNPs from the majority of isolates, were not part of the outbreak. A further two isolates, separated by more than 30 SNPs from the majority of isolates, were re-typed and found to not match the E1244 MIRU-VNTR outbreak profile. Isolates from 344 individual patients were retained for outbreak analysis.

### Outbreak Diversity

**Fig 2:**
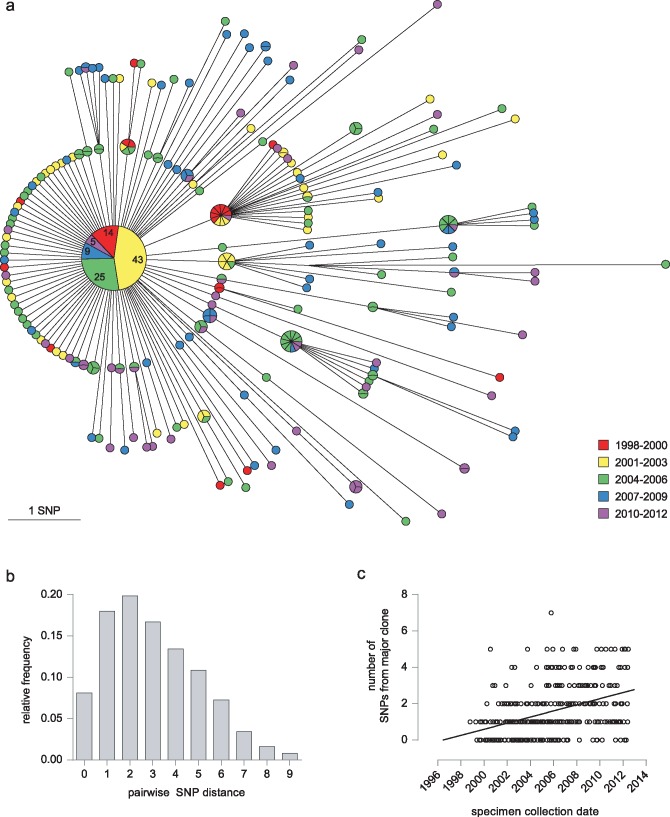
Outbreak diversity. (a) MST of 344 isolates from individual patients. Colour coding of nodes indicates the year of isolation. Each isolate is represented by a separate segment, except for the central major clone, where the number of isolates from each time period is given. (b) Histogram illustrating pairwise SNP distances between isolates. (c) Graph showing date of specimen collection against its SNP distance from the major clone.

By whole genome sequence analysis of SNPs, 96 (27.9%) of the 344 isolates were indistinguishable. A further 105 (30.5%) differed from this major clone by a single SNP, and only one isolate differed from the major clone by more than five SNPs (Fig 2A). The maximum number of SNPs between any pair of isolates was nine; the modal distance between isolates was two SNPs (Fig 2B), and the mean distance was 3.03 SNPs (±2.03). Overall, 270 SNPs discriminated isolates within the cluster. Accumulation of SNPs showed a weak temporal trend (0.18 SNPs/year) and was consistent with introduction of the clone into the UK population in the mid-1990s (Fig 2C). The data implicated a unique source case for only 16 (4.7%) cases, and the longest chain of transmission derived was three cases (S1 Fig). All deduced directional links were consistent with the collection dates of the isolates.

The effect of including variant sites in the PE-PPE genes, in which long repeat regions with a high GC content mean SNP calls may be less robust (see Methods), is shown in S2 Fig.

### Correlation with Epidemiological Investigations

A detailed epidemiological investigation describing links between the first 79 cases, identified between 1995 and 2001, was published by Ruddy et al. [1]. Sequences of 60 of these patients’ isolates were available. Of these, 33 belonged to the major clone; there was also a cluster of seven indistinguishable isolates and three clusters of two.

**Fig 3:**
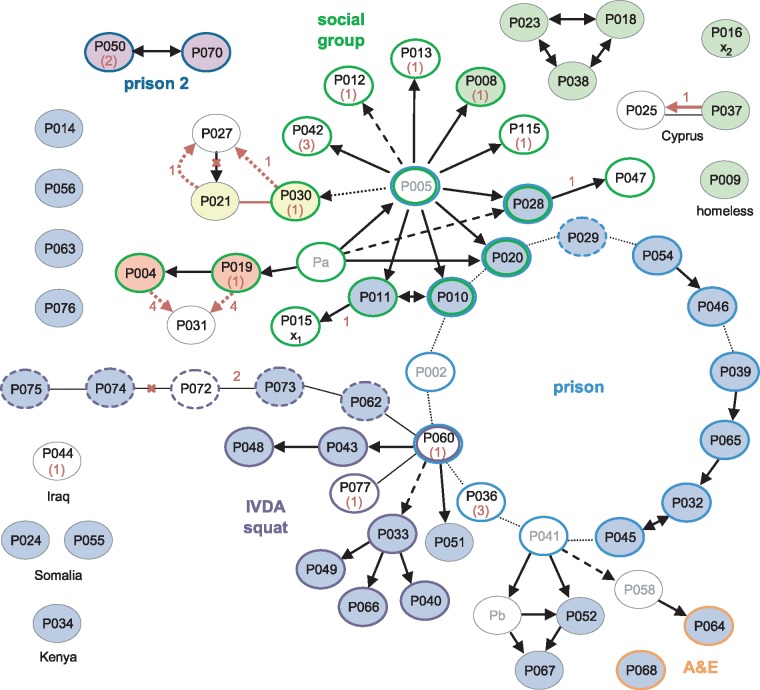
Correlation of epidemiological data with WGS. Black arrows and lines joining ovals indicate links derived from contact tracing, with solid, dashed, and dotted lines indicating increasing levels of uncertainty [1]. For clarity, unlinked cases without sequences are omitted; linked cases without sequences or without isolates (Pa-b) are in grey font. The source and recipient of a putative laboratory cross-contamination are marked x_1_ and x_2_, respectively. Coloured oval outlines group-associated cases. Green outlines define a group of socially linked young adults in North London. Blue outlines indicate individuals detained at the same prison; the dashed line is a staff member at the prison, and dark blue lines indicate a different prison. Purple outlined cases were linked to a squat in North London, with dashed lines possibly also linked. Orange outlines indicate staff in hospital departments through which many of the smear-positive patients had passed. Isolates that were indistinguishable by WGS are filled with the same colour; blue-filled ovals belong to the major clone. Numbers in red represent the number of SNPs separating isolates. Where the sequence of the putative source case isolate is unavailable, the SNP distance from the major clone is given in brackets. Red arrows and lines signify transmission events suggested by the WGS data. Red crosses indicate epidemiological links refuted by the WGS data.

The earliest case, diagnosed in 1995, and 18 other cases were socially linked through a group of young adults in the North London area (Fig 3). Amongst the 14 of these isolates that were sequenced, four belonged to the major clone, and a further nine distinct genotypes were present (S3 Fig). Three of the group, who all harboured the major clone, had been detained in a London prison, where a further 11 outbreak cases were identified, including one staff member (P029). A total of 12 of the 14 sequenced prison-associated isolates belonged to the major clone.

A third group of cases was linked via a North London squat frequented by intravenous drug abusers (IVDA) and to the prison cluster through P060. Ten of the 12 further patients that were strongly or possibly linked to the squat were infected with the major clone. The sequenced isolate from P060 was obtained in 2001 and differs from the major clone by a single SNP encoding the rifampicin-resistance mutation RpoB H445R. The phenotype of an isolate obtained in 2000 from this patient was rifampicin-sensitive, suggesting that he was originally infected with the major clone through prison contacts and may have transmitted the clone within the squat before acquiring rifampicin resistance.

The WGS data suggested several links that had not been uncovered through epidemiological investigations (P021 and P030; P004 or P019 and P031), and a shared genotype linked seven cases, only three of which were linked epidemiologically. However, the possibility of intervening unidentified cases cannot be discounted. The data revealed the direction of transfer in one instance (P037 to P025) and refuted the direction in another (P027 to P021).

One of the isolates, belonging to a retired woman with no obvious links to the other groups (P016), was strongly suspected to be a cross-contamination from an isolate processed in the same laboratory on the same day (P015) [1]. These two isolates each differ from the major clone by one unique SNP. Furthermore, the putative contaminant belonged to the cluster of seven identical isolates, making the cross-contamination theory less plausible (S3 Fig).

### Acquisition of Drug Resistance and Compensatory Mutations

Thirteen isolates from 11 patients were resistant to rifampicin, in addition to isoniazid, and were thus multidrug-resistant (MDR) (Table 1). Three of the isolates had unique substitutions in RpoB codon 445. The remaining isolates harboured rare resistance-conferring mutations in codon 170 [20,21]; seven isolates carried V170F, and in one isolate, a second mutation within the codon resulted in the substitution F170L. This distribution of genotypes suggests that four patients acquired rifampicin-resistance and seven had primary MDR-TB. Whilst homoplasic acquisition of drug resistance SNPs is common, the rarity of V170F [22] supports its transmission.

**Table 1:** Drug resistance and compensatory mutations in poly-resistant isolates^a^.

Isolate	Patient	INH	P_*inhA*_	RIF	RpoB	RpoA	RpoC	PZA	*pncA*	EMB	STR
M02010519	P052	R	-15 c > t	R	V170n^b^	.	.	S	.	S	S
M04001224	P052	R	-15 c > t	R	V170F	.	W484G	S	.	S	S
M04000819	P153	R	-15 c > t	R	V170F	T187A	.	S	.	S	S
M05010916	P224	R	-15 c > t	R	V170L	.	N698S	S	.	S	S
M05011996	P230	R	-15 c > t	R	V170F+T399I	.	.	R	170 a > at	S	S
M08003689	P316	R	-15 c > t	R	V170F	.	G571R	S	.	S	S
H121920020	P316	R	-15 c > t	R	V170F	.	G571R	S	.	S	S
H101480093	P360	R	-15 c > t	R	V170F	.	G571R	S	.	S	S
H102780066	P367	R	-15 c > t	R	V170F	.	G571R	S	.	S	S
H111560023	P367	R	-15 c > t	R	V170F	.	G571R	S	.	S	S
H112020004	P383	R	-15 c > t	R	V170F	.	G571R	S	.	S	S
M01004483	P060	R	-15 c > t	R	H445R	.	.	S	.	S	S
M07011246	P251	R	-15 c > t	R	H445D	.	.	S	.	S	S
M10010295	P356	nd	-15 c > t	nd	H445N	.	.	nd	.	nd	nd

^a^ Phenotypic resistance (R) or sensitivity (S) to isoniazid (INH), rifampicin (RIF), pyrazinamide (PZA), ethambutol (EMB), and streptomycin (STR), with associated genotypes in the *mabA-inhA* operon promoter (P_*inhA*_, INH), RpoB (RIF), and PncA (PZA). Substitutions in RpoA or RpoC and RpoB T399I are putative compensatory mutations. Nucleotides are given in lower case and amino acids in upper case. nd, not determined.

^b^ Heterogeneous allele (see Acquisition of Drug Resistance and Compensatory Mutations).

Isolates with rifampicin-resistance mutations frequently harbour fitness compensatory mutations within the *rpoABC* genes [12,23]. Remarkably, amongst the isolates carrying mutations in codon 170, secondary mutations in *rpoABC* genes were independently acquired at least five times, strongly suggesting they are the result of positive selection and supporting a role in fitness compensation.

**Fig 4:**
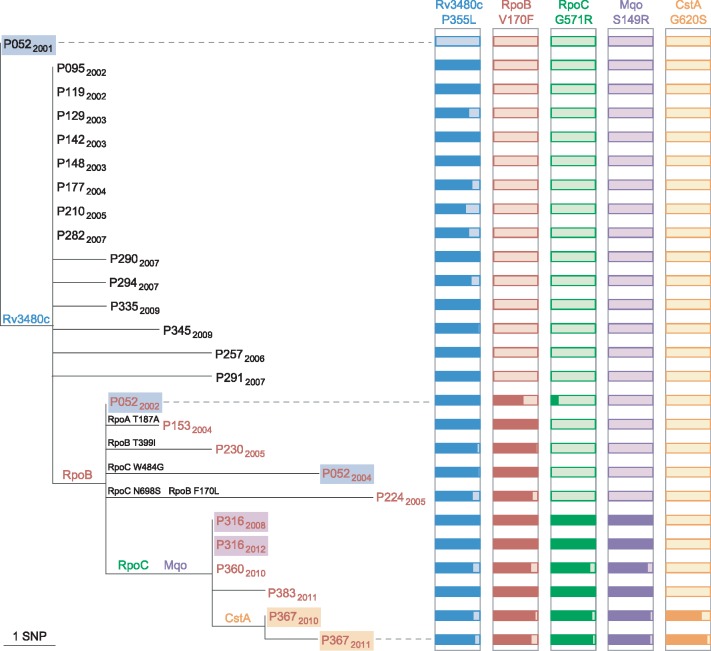
Allele heterogeneity associated with clustered MDR cases. Maximum likelihood tree of the phylogenetic clade containing isolates with the RpoB V170F substitution. The isolate from P052 in 2001 belongs to the major clone. Isolates from the same patient are highlighted, and those with a rifampicin-resistant phenotype are in red font. For each of the five SNPs that are shared by isolates in the clade, histograms illustrate the proportion of reads that match the mutated allele (dark coloured). Additional mutations within RpoABC are also shown on tree branches.

Public health investigators identified a patient with poor adherence, P052, as the source of the clustered MDR cases [24]. Sequencing of three isolates from this patient revealed the evolution of rifampicin resistance from a homogeneous wild-type allele in April 2001, through a heteroresistant genotype (with 66% of reads matching the mutant base) in November 2002, to a homogeneous mutant allele in February 2004 (Fig 4). Phenotypic drug susceptibility testing was concordant with the predominant genotype. Investigation of heterogeneous basecalls within the *rpoABC* genes revealed that the second isolate carried a minority variant RpoC G517R. This mutation was identified in four of the other MDR cluster patients’ isolates but was not detectable in the third isolate from P052, which carried a homogeneous substitution encoding RpoC W484G. These results are consistent with the contact tracing data and demonstrate acquisition of rifampicin resistance by P052 and onward transmission of the intermediate genotype RpoB V170F RpoC W484G that P052 harboured in 2002.

### Intra-host Diversity

In order to further investigate within-host diversity, we selected pairs of patient isolates obtained from pulmonary sites more than 6 mo apart. These patients remained culture-positive, reflecting that the treatment of many of the patients was challenging, as evidenced by the development of MDR-TB in a small number of patients. As Menzies et al. [25] pointed out in their systematic review of treatment for isoniazid-resistant tuberculosis, treatment failure rates and relapse rates are much higher than previously thought.

**Fig 5:**
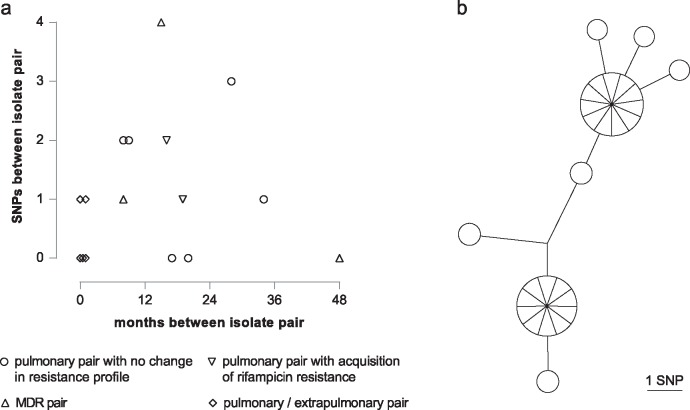
Intra-host diversity. (a) Graph illustrating SNP distance between pairs of patient isolates. (b) MST of 27 individual colonies isolated from a single sputum specimen.

Six pairs isolated 9 to 35 mo apart differed from each other by between zero to three SNPs (Fig 5A). For two patients who acquired rifampicin resistance, both sensitive and resistant isolates were available. In addition to the rifampicin resistance conferring SNP, one isolate fixed a second mutation, whilst the other did not. Three pairs of pulmonary MDR isolates were identified; one pair obtained four y apart were identical, whilst the other two separated by 8 and 15 mo differed by one and four SNPs, respectively. Based on the hypothesis that extra-pulmonary dissemination would constitute an evolutionary bottleneck resulting in fixation of minority variants, we sequenced five contemporary pairs of pulmonary and extrapulmonary isolates. Three pairs were identical, and two were separated by a single SNP.

For routine culture of archived isolates for WGS, an aliquot was plated and, after incubation, sweeps of colonies or confluent growth were harvested for DNA extraction. This procedure ensures that at each genome position the base called corresponds to the major allele in the population. In order to assess the frequency of minor variants in a sample, we cultured the residual pellet from a decontaminated sputum specimen, picked 27 of the resulting colonies, and sequenced them individually. Ten loci differed amongst the colonies, with a maximum distance between any pair of six SNPs (Fig 5B).

### Comparison of WGS with Molecular Fingerprinting

The outbreak is defined by a characteristic 15-band RFLP fingerprint, LAM10 spoligotype (777777743760771), and 24-loci MIRU-VNTR profile (PHE cluster E1244). The VNTR profile includes a single locus (3690) that is characteristically untypeable for all isolates within the cluster. Examination of the number of sequencing reads mapping to this locus indicated that the untypeability was due to the large number of repeats at this locus, in agreement with previous reports (S4 Fig) [26].

In order to compare WGS-based clustering with MIRU-VNTR–based clustering, 14 isolates that differed from the canonical E1244 profile at 1 or 2 loci or had incomplete typing data were sequenced. Comparison of these variant isolates with the outbreak sequences showed that the isolates with a polymorphic number of repeats at one locus differed from their closest outbreak sequence by zero to three SNPs and clustered within the outbreak phylogeny, whereas isolates that were polymorphic at two VNTR loci differed by 32–34 SNPs from the closest neighbouring E1244 sequence (Table 2; S5 Fig). The VNTR variants that fell within the outbreak cluster all carried its characteristic isoniazid-resistance mutation in the *inhA* promoter, whereas those outside the cluster did not.

**Table 2:** Comparison of VNTR-based clustering with WGS.

	VNTR locus^a^																								SNP distance to neighbour^b^	Isoniazid resistance genotype	Rifampicin resistance genotype
	A	B	C	D	31	2	10	16	20	23	24	26	27	39	40	424	1955	2163b	2347	2401	3171	3690	4052	4156			
**E1224**	4	2	4	3	3	2	4	3	1	5	1	5	3	2	1	2	3	6	4	2	3	-	5	2			
H104840010	4	2	4	3	3	2	3	3	1	5	1	5	3	2	1	2	3	6	4	2	3	-	3	2	32	KatG S315T	RpoB H445D
H112540239	4	2	4	2	3	2	3	3	1	5	1	5	3	2	1	2	3	6	4	2	3	-	5	-	34	-	-
H120180038	4	-	-	3	3	2	-	3	1	5	1	-	-	2	1	2	-	6	4	2	3	-	5	-	43	-	-
M09006047	4	2	4	-	3	-	-	3	1	5	-	5	3	2	1	2	3	6	4	2	3	-	5	2	0	P_*inhA*_ -15 c > t	-
M10010190	4	2	4	3	3	2	4	3	1	5	1	5	-	2	1	2	3	6	-	2	3	2	5	2	0	P_*inhA*_ -15 c > t	-
M10011453	4	2	4	3	3	2	4	3	1	5	1	5	3	2	1	2	2	6	4	2	3	-	5	2	1	P_*inhA*_ -15 c > t	-
H103220122	4	2	4	3	3	2	4	3	1	5	1	5	3	2	1	2	3	3	4	2	3	-	5	2	2	P_*inhA*_ -15 c > t	-
H103560010	4	2	4	3	3	2	4	3	1	5	1	5	3	2	1	2	3	6	4	2	3	-	4	2	0	P_*inhA*_ -15 c > t	-
H110180002	4	2	4	3	3	2	4	3	1	5	1	5	3	2	1	2	3	5	4	2	3	-	5	2	1	P_*inhA*_ -15 c > t	-
H110380011	4	2	4	3	3	2	4	3	1	5	1	5	3	2	1	2	3	5	4	2	3	-	5	2	0	P_*inhA*_ -15 c > t	-
H111900033	4	2	4	3	3	2	-	3	1	5	1	5	3	2	1	2	3	6	4	2	3	-	3	-	2	P_*inhA*_ -15 c > t	-
H130740103	4	2	4	3	3	2	4	3	1	5	1	5	3	2	1	2	3	6	4	2	3	-	4	-	1	P_*inhA*_ -15 c > t	-
H133320003	4	2	4	3	3	2	4	3	1	5	1	5	3	2	1	2	3	6	4	2	2	-	5	2	1	P_*inhA*_ -15 c > t	-
H133440013	4	2	4	3	3	2	4	3	1	5	1	5	3	2	1	2	3	4	4	2	3	-	5	2	3	P_*inhA*_ -15 c > t	-

^a^ Dashes indicate missing data; variant repeat numbers are underlined.

^b^ Closest E1244 sequence.

VNTR, variable number tandem repeat; SNP, single nucleotide polymorphism.

Spoligotypes derived from sequencing data indicated that a number of isolates differed from the canonical type by the loss of spoligotype spacer 19. The derived profiles were confirmed by laboratory-based typing of representative isolates. Mapping the deletion onto the SNP-derived phylogeny suggests homoplasic loss. However, deletion prior to acquisition of SNPs is a more parsimonious explanation and provides a link between these patients (S5 Fig).

### Outbreak-Defining Polymorphisms

Outbreak-specific polymorphisms were defined as those present in all E1244 sequences and absent from the three isolates that were separated from the major clone by 32–43 SNPs (Table 2). Thirteen outbreak-defining SNPs included eight encoding nonsynonymous substitutions and two promoter mutations. Four of the nonsynonymous SNPs affected genes involved in lipid metabolism or peptidoglycan synthesis and could promote virulence through altering properties of the cell wall.

Outbreak isolates shared a 1 bp deletion within Rv1364c, an anti-sigma factor antagonist [27], which results in truncation of the protein product from 675 to 376 amino acids. No structural variants characterising the outbreak were detected.

## Discussion

To our knowledge, this is the largest WGS-based study of an outbreak of *M*. *tuberculosis* reported to date. Analysis of 344 isolates, sharing an identical 24-loci VNTR profile molecular type, collected from individual patients over 14 y, revealed that 96 (38%) were indistinguishable, and only one differed by more than five SNPs from this clone. Contrary to that observed in some recent studies, WGS was able to reveal the direction of transmission of tuberculosis in only a small proportion (16/344) of cases within the outbreak. We observed the acquisition and spread of a rare RpoB mutation conferring rifampicin resistance that supported the conclusions of a public health investigation into this MDR cluster [24]. Despite the overall lack of variation in isolates between patients, intra-patient isolate diversity was detected in longitudinal isolates (up to 4 SNPs in 4 y), across specimen sites (up to 1 SNP in contemporary isolates), and within a single specimen (10 SNPs in 27 individual colonies).

The relative lack of between-patient diversity compared to within-specimen diversity might suggest that transmission may not involve a narrow evolutionary bottleneck, or that the population typically involves one very abundant clone and multiple rare or minor clones. However, we speculate that where low numbers of bacilli are transmitted, such as during casual contact, variants existing in the source donor population become fixed in the recipient due to the founder effect. Neely et al. [6] showed that the highest transmission rate in this outbreak was among contacts exposed to two or more cases; this is likely to have occurred among the prison-associated cases (unfortunately, institutional contact tracing was limited) and may contribute to the almost complete lack of genetic diversity within this group. The concept of a transmission chain or network may not always be a useful model in institutional or household settings where concurrently infectious cases are continuously exchanging bacilli rather than a single infectious index case. In this scenario, the cases would be expected to share the same dominant genotype, as we observed. In contrast, for the group related by casual social contact, a single transmission event is consistent with the divergence observed between the source and recipient cases.

Based on studies in the low burden settings of Oxfordshire and the Midlands in the UK, Walker et al. [28,29] propose a 12-SNP distance as the gold standard upper threshold to identify plausible transmissions and argue that isolates separated by five or fewer SNPs are likely to have resulted from recent transmission. Our data indicate that in this London outbreak, multiple transmission events can occur with no detectable SNP acquisition, so that even identical isolate pairs cannot be deduced to have resulted from a direct transmission event without a supporting epidemiological link. On the other hand, our data support the premise that WGS alone can be used to rule out the possibility of direct transmission when isolates are separated by a substantial number of SNPs. Taken together, our data suggest that WGS may not be as useful in identifying the direction of transmission as previously supposed.

The level of diversity observed in this outbreak, averaging 0.8 SNPs per case (270 SNPs/344 isolates) is slightly lower than that found in other similar studies of large community clusters. A Hamburg cluster had an average of 1.0 SNPs per case (86 SNPs/85 isolates) [30], a Toronto cluster had 1.5 SNPs per case (81 SNPs/55 isolates) [31], and a Bernese cluster had 2.0 SNPs per case (133 SNPs/68 isolates) [32]. The lack of variation observed in the London outbreak may be due to its introduction into vulnerable populations with risk factors for transmission and poor adherence to therapy, maintaining infectivity and transmission. Alternatively, it could be due to properties of the clone.

The VNTR 3690 locus is located upstream of *lpdA* (Rv3303c), encoding a flavoprotein disulfide reductase that has been shown to protect *M*. *tuberculosis* from oxidative stress and contribute to enhanced survival in a mouse model [33]. Variation in the number of VNTR 3690 repeats affects the magnitude of *lpdA* expression [34]; thus, it is possible that the high copy number in the outbreak isolates contributes a virulent phenotype to this strain. Intriguingly, Rv1364c, which is truncated in the outbreak strain, is located within a 15 kb deletion that characterises the dominant homogenous sub-cluster of the ON-A strain that has been endemic in the homeless population of Toronto, Canada, for over 17 y [31]. Rv1364c is a regulator of the alternative sigma factor SigF [27], which is postulated to modulate immunopathogenesis in the infected host by modifying the cell envelope [35].

It is remarkable that of the four acquisitions of rifampicin-resistant genotypes within the outbreak reported here (RpoB V170F, H445D, H445N, H445R) plus two reported previously (RpoB D435Y, S450W) [21,24], none are the S450L mutation that dominates the rifampicin-resistant population. This observation is suggestive of an epistatic effect, but no candidate causal polymorphisms were identified in RNA polymerase subunit genes.

One limitation of the study is that investigation of intra-specimen diversity was only possible for one isolate, and the results may not be more generalizable. However, the data suggest that the practice of isolating single colonies prior to sequencing is likely to lead to an overestimation of the number of SNPs between cases resulting from direct transmission. Our approach, in which the sequence of the patient’s dominant genotype is determined, is preferable. A further limitation is that variation in repetitive regions, amounting to almost 10% of the genome, was not assessed due to the difficulty in reliably calling SNPs within these regions using current technologies (as described in Methods). Similarly, unambiguous data were not obtained for indels. In the future, this problem will be ameliorated by the availability of longer high-quality reads. Development of analysis workflows that incorporate a systematic and reliable analysis of repeat regions, small insertions, and large sequence polymorphisms may reveal some further variation within the outbreak.

However, studies have cast doubt on the previously widely held belief that the PE-PPE genes contain a large amount of variation [36,37]. Although some studies using strains from across the whole diversity of *M*. *tuberculosis* have suggested that the variation in the PE-PPE genes is 3-fold higher than in non-repetitive genes [38,39], this would not generate sufficient extra variation within closely related isolates from a transmission chain to allow accurate determination of transmission. Similarly, even if including short insertions/deletions allowed us to capture an extra 10% of variation on top of this, it would still not provide enough variation to invalidate our main conclusion that there is insufficient variation within the outbreak to confidently call transmission chains.

WGS studies of large tuberculosis outbreaks, including this one, have typically been directed by VNTR- or RFLP-based clustering of isolates [30–32]. In this study, all isolates that matched the 24-loci E1244 VNTR cluster profile also belonged to the outbreak by WGS-based clustering and those differing at two loci did not, indicating that, for this dataset, requiring a complete 24-loci match for clustering underestimates the true outbreak size. However, the discriminatory power of standard 24-loci VNTR-typing varies between *M*. *tuberculosis* lineages and sublineages [40], and this may not be true across the species. Population-based comparisons are required to assess the true power of VNTR-based clustering to reflect WGS-linked cases in different settings.

We conclude that whilst WGS provides increased resolution over VNTR-based clustering, without supporting epidemiological data, WGS is insufficient to resolve transmission networks in tuberculosis outbreaks. Furthermore, the data suggest that the concept of a transmission chain or network may not be useful in institutional or household settings. The practice of applying a fixed SNP distance alone to identify plausible transmissions needs to be considered within the context of the specific population. WGS technology remains useful in ruling out the possibility of direct transmission when isolates are separated by a substantial number of SNPs. Although the value of WGS in defining drug resistance and identifying compensatory mutations was supported in this study, the real value of wide-scale investment in WGS to understand tuberculosis transmission in the context of public health action needs to be very carefully considered in the light of the above findings.

## Supporting Information

S1 Fig MST illustrating inferred transmission links. MST of 344 isolates from individual patients. Colour coding of nodes indicates the year of isolation. Each isolate is represented by a separate segment except for the central major clone, for which the number of isolates from each time period is given. Branches coloured red highlight links where a case can be linked to a single source case. The inferred node, indicated by the red unfilled circle, is occupied by the second isolate of a patient series.
(EPS)

S2 Fig MST of isolates including SNPs within PE-PPE genes. (a) MST of 344 isolates from individual patients. Colour coding of nodes indicates the year of isolation. Each isolate is represented by a separate segment except for the central major clone, for which the number of isolates from each time period is given. (b) Histogram illustrating pairwise SNP distances between isolates. (c) Graph showing date of specimen collection against its SNP distance from the major clone.
(EPS)

S3 Fig MST of 60 isolates from cases with epidemiological data. Nodes represent the 60 sequenced isolates shown in Fig 3 and are colour-coded by the location of patients’ interaction. x_2_ marks the case suspected to be a cross-contamination from case x_1_.
(EPS)

S4 Fig Mapping of reads to the untypeable VNTR locus 3690. Line graphs, generated by the bam-viewer Artemis, show the number of sequencing reads mapping to the H37Rv reference genome, which has two copies of the 3690 repeat.
(EPS)

S5 Fig MST illustrating VNTR and spoligotype polymorphisms relative to WGS. Nodes of isolates that differ from the E1244 VNTR profile or the canonical LAM10 spoligotype are coloured. Branch lengths are logarithmically scaled.
(EPS)

S1 Text Prospective analysis plan. (PDF)

## Abbreviations

IVDA: intravenous drug abusers
MDR-TB: multidrug-resistant tuberculosis
MIRU-VNTR: mycobacterial interspersed repetitive units-variable number tandem repeats
MST: minimum spanning tree
PHE: Public Health England
RFLP: restriction fragment length polymorphism
SNP: single nucleotide polymorphism
WGS: whole genome sequencing
