# Supplementary material for: Whole Genome Sequence Analysis of a Large Isoniazid-Resistant Tuberculosis Outbreak in London: A Retrospective Observational Study
Source: PLoS Med. 2016 Oct 4;13(10):e1002137. doi: 10.1371/journal.pmed.1002137 (PMC5049847; doi:10.1371/journal.pmed.1002137)
Supplement: S1 Text — (PDF) [file pmed.1002137.s006.pdf]

PRELIMINARY APPLICATION FOR PATHOGEN SEQUENCING PROJECT MANAGEMENT MEETING

Reference number:

- This form should only be used for projects related to the Pathogen-Variation Programme working group.
- Please send the completed form to Sophie Palmer (sophie@sanger.ac.uk) and Bridget Jasper (bj3@sanger.ac.uk).
- Please complete all sections and relevant drop-down boxes.

SECTION A: GENERAL INFORMATION

1. Name of WTSI project manager

Julian Parkhill

2. Date

30/10/2012

3. SAC sponsor

4. In collaboration with (name, institution and contact details)

Francis Drobniewski
Queen Mary, University of London
f.drobniewski@qmul.ac.uk

5. Species and size of genome

Mycobacterium tuberculosis
4.4Mb

6. Project title

Tuberculosis outbreak investigation

7. Description of the project, including a summary of the scientific merits, outcomes or goals and concepts which form the basis of the proposal, the stated aims, and tasks to be performed at Sanger (~1 page)

We propose to sequence isolates belonging to a large and ongoing outbreak of isoniazid-monoresistant tuberculosis (TB) that was first identified in 2000 and is centred on North London. Clustered isolates were identified retrospectively and prospectively at the HPA National Mycobacterium Reference Laboratory (NMRL) using IS6110 RFLP or, more recently, MIRU-VNTR. Between 1998-2012 over 400 cases belonging to the cluster have been identified in North London and it is thought to be the largest documented outbreak of drug-resistant TB in Europe so far (Maguire et al, 2011, Eurosurveillance 16:13).

Epidemiological data, obtained through questionnaires, on the first 100 confirmed cases established social links between groups of patients and in some instances chains of transmission have been deduced (Ruddy et al, 2004, Thorax 59:279). An unusually high rate of transmission to contacts of infectious patients has been reported (Neely et al, 2010, J. Public Health 32:44).

We plan to sequence all isolates from outbreak cases that are archived at the NMRL, a total of about 475. This includes approximately 10 isolates that are multi-drug resistant, that is resistant to isoniazid plus rifampicin.

Given that almost nothing is known about the genetic variation within an epidemiologically-linked VNTR cluster, in order to put our findings into perspective, we propose to also investigate the degree of diversity within a single specimen and within the human host. To assess intra-specimen variation, we will plate a bacterial pellet recovered from a

decontaminated sputum sample from an outbreak case. DNA will be isolated from 96 individual colonies, without further subculture, to minimise possible in vitro selection. For about five cases, serial isolates obtained over the course of therapy are available (3mo to 1yr between isolates). These include pulmonary and extrapulmonary specimens from the same patient. Sequencing of these isolates will enable us to investigate within host evolution during the course of an infection.

Sequencing the genomes of M. tuberculosis isolates from an outbreak spanning 15 years affords a unique opportunity to study the evolution of this strain within a community. Furthermore, this project will provide much needed data to inform the debate on the utility sequencing of whole genome sequencing as a routine typing tool. The data will enable us to explore:

- the rate of mutation
- diversity within and between hosts
- genetic determinants of epidemiological success
- genetic bottlenecks associated with drug resistance, dissemination to extrapulmonary sites and transmission
- the importance of natural selection versus genetic drift
- correlation between molecular fingerprint-derived clusters and SNP clusters
- whether the direction of transmission between contacts can be determined or superspreaders identified

8. Anticipated project start date

December 2012

9. Anticipated date samples available in-house

December 2012

11. Will you be using the data coordination team? (DNA Pipeline services 1 team184)

10. Project duration (months) or end date

12
